# Historical control data for the interpretation of ecotoxicity data: are we missing a trick?

**DOI:** 10.1007/s10646-019-02128-9

**Published:** 2019-11-06

**Authors:** Amy C. Brooks, Manousos Foudoulakis, Hanna S. Schuster, James R. Wheeler

**Affiliations:** 1 Cambridge Environmental Assessments, Cambridge, UK; 2 Corteva Agriscience™, Agriculture Division of DowDuPont™, Abingdon, UK; 3 Present Address: Shell International B.V. Shell Health, Carel van Bylandtlaan 16, 2596 HR The Hague, The Netherlands

**Keywords:** Historical control data, Ecotoxicology, Variability, Endpoints, Regulatory

## Abstract

Wildlife can be exposed to chemicals in the environment from various anthropogenic sources. Ecotoxicity studies, undertaken to address the risks from potential exposure to chemicals, vary in their design e.g. duration of exposure, effect types and endpoints measured. Ecotoxicity studies measure biological responses to test item exposure. Responses can be highly variable, with limited opportunity for control of extrinsic sources of variability. It is critical to distinguish between treatment-related effects and background ‘normal variability’ when interpreting results. Historical control data (HCD) can be a valuable tool in contextualising results from single studies against previous studies performed under similar conditions. This paper discusses the case for better use of HCD in ecotoxicology assessments, illustrating with case studies the value and difficulties of using HCD in interpretation of results of standard and higher-tier study designs. HCD are routinely used in mammalian toxicology for human health assessments, but not directly in ecotoxicology. The possible reasons for this are discussed e.g., different data types, the potential to mask effects, and the lack of guidance. These concerns are real but not insurmountable and we would like to see organisations such as OECD, EFSA and USEPA develop guidance on the principles of HCD collection. Hopefully, this would lead to greater use of HCD and regulatory acceptance. We believe this is not only a scientifically valid approach but also an ethical issue that is in line with societally driven legal mandates to minimise the use of vertebrate testing in chemical regulatory decision making.

## Introduction

### What’s the regulatory problem?

Wildlife can potentially be exposed to chemicals in the environment from various anthropogenic sources, e.g., plant protection products, biocides, veterinary medicines and pharmaceuticals. Risk assessments are undertaken in accordance with the relevant legislative frameworks (e.g. EC 2009) to determine uses that will be protective of species in the environment. To estimate the risk, potential exposure and hazard are determined. To address the latter, ecotoxicity studies are undertaken, using standard species and defined test guideline methods (e.g. U.S. EPA 2018, OECD 2018).

Ecotoxicity studies determine what effects may be induced in either short term acute (generally lethality) or long term chronic (sublethal, e.g., reproduction, growth, development) exposures. The responses of organisms being exposed to a chemical (‘treated’) and the responses of organisms in the absence of the chemical (the concurrent ‘controls’) are used to determine different types of endpoints (Box 1).

#### Box 1 Toxicity endpoints

There are three main types of toxicity endpoint that can be derived for a test species exposed to a particular chemical:

1. *Exposure level at which a particular percentage of effect occurs*, commonly referred to as EC_x_, where *x* can range between 0 and 100%, but is typically 10%, 20% or 50%. The derivation of an EC_x_ endpoint requires that a dose-response is observed, ideally with effects observed in the study covering the full range from 0% to 100% effect to allow an accurate dose-response regression curve to be plotted and thus a reliable estimate of the EC_*x*_ to be calculated.

2. *Exposure level at which the lowest level of detectable effect is observed*, commonly referred to as LOEC (lowest observed effect concentration). The LOEC is the lowest tested concentration at which a statistically significant treatment-related effect (typically at *p* < 0.05) is observed. However, all test levels above the LOEC should have a harmful effect equal to or greater than those observed at the LOEC. The next lowest tested concentration is the NOEC (see below). Therefore the LOEC is influenced by the spacing between the concentrations tested.

3. *Exposure level at which no effect was observed*, commonly referred to as NOEC (no observed effect concentration). The NOEC is the highest tested concentration at which no statistically significant treatment-related effects (typically at *p* < 0.05) are observed, with the next highest tested concentration being the LOEC (see above). Therefore the NOEC is influenced by the spacing between the concentrations tested. Consequently, some support the use of the No Effect Concentration (NEC; Kon et al. 2015).

For all of these types of endpoints, the responses of organisms in the replicates treated with the test chemical are compared to the responses of organisms in the negative control(s) (i.e. in the absence of test chemical).

A hypothetical example of how these endpoints are derived is presented in the graph below. An important point to note from the graph is that there is variability (standard error bars in this example) around each of the treatments means for a specific biological response are due to natural variation, including in the control group. It is this variation that can be contextualised using historical control data.


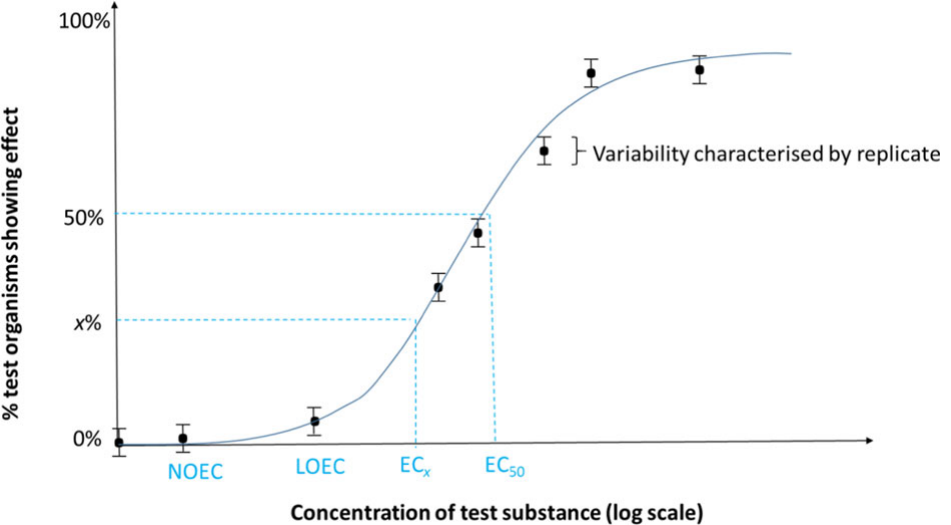


The design of toxicity studies depends on the type of effects that are being investigated and the organisms being used. Studies investigating sublethal effects are typically of a longer duration, and tend to require more sampling occasions and more organisms than shorter term lethal effect studies. Sublethal studies have traditionally aimed to determine a No Observed Effect Level or Concentration (NOE(L)C), but increasingly both NOELs and Effective Concentration inducing a specified percent effect (ECx) determinations are being required (EFSA 2015). Whilst there is growing support for the use of the No Effect Concentration (NEC; Kon et al. 2015). The optimal design for either a hypothesis or regression based statistic is different (Green et al. 2012); however, both require assessments over different treatment levels (concentrations, doses) in order to adequately capture the threshold or response curve for a particular effect. Consequently, ecotoxicity studies typically employ at least 5 test item treatment levels. In some circumstances, certainly where vertebrates are employed, efforts are made to reduce the number of levels (e.g., avian reproduction, fish and amphibian endocrine screening studies) so designs are more akin to mammalian toxicology test designs. The complexity of a study is also a factor, with high numbers of replicates and treatment levels often being impractical and too costly for higher-tier studies (e.g., mesocosms). Having fewer replicates and treatment levels in a study design means that less information is available on the full extent of the variability in response in both the control and treated groups. Variability can be particularly apparent in sublethal effect studies due to the nature of the parameters being observed (i.e. there is more of gradient of effect as well as multiple responses).

**Fig. 1:**
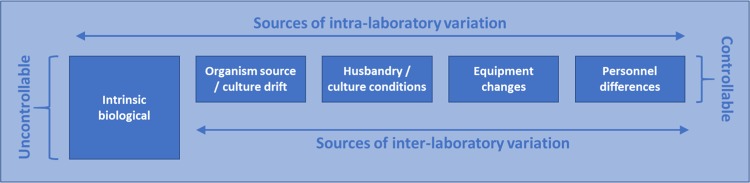
Sources of variation within standard ecotoxicological tests

Ecotoxicology tests are essentially measuring the biological response of an organism to the test item. However, this response will always be a variable measure of some biological process under stress. Consequently, standardised methods aim to reduce this variability as far as possible both within laboratories and amongst laboratories (see Fig. 1). However, it should be recognised that the intrinsic biological variability of a response cannot be controlled. For some parameters, this can be a significant proportion of the overall variability of the test system. For example, Valverde-Garcia et al. (2018) found that the variation within a study (intrinsic biological variability and typical experimental variation at the laboratory) accounted for the majority (64.9–93.4%) of the variability in responses in avian reproduction studies. Unfortunately, some of the most variable parameters are often considered the most important to the protection goals of environmental risk assessment (e.g. reproduction). To a large extent, the development of internationally recognised test guidelines (e.g., OECD) aim to address this problem by providing appropriate test designs (e.g., number of treatments, replication) and statistical approaches to determining biologically relevant effects. This may be statistically achieved by establishing thresholds of effects (i.e. hypothesis testing) or determining an appropriate percent effect (i.e., regression testing). For either to be meaningful they must distinguish treatment related effects against the background of ‘normal variability’.

One of the challenges of variable responses is dealing with the wide range of ‘normal’ outcomes and its impact on statistical power. If, by chance, the control replicates are mainly comprised of organisms that tend to exhibit responses at the high end of ‘normal’, and a treatment group is mainly comprised of those exhibiting responses at the lower level of the ‘normal’ range, a statistically significant decrease compared to the concurrent control could result, but this would be highly likely due to chance alone rather than a true treatment effect. Such outcomes are potentially easier to interpret if the statistically significant difference occurs at a low or intermediate treatment, with no apparent treatment-response relationship, but can be more difficult where it occurs at the highest treatment tested. Another potential consequence of variable responses is that trends can occur across treatment groups, which may not be statistically significantly different to the concurrent control but appear to be biologically relevant and thus are conservatively considered treatment related. This is clearly the result of not having a treatment at a sufficiently high level to induce a real effect. It can therefore be challenging to interpret the results from sublethal effect studies, where responses are variable and it is not possible to dramatically increase the number of treatments or replication to better describe this variability, particularly in the case of vertebrate studies, where there is the need to minimise the number of vertebrates as far as possible (Burden et al. 2015).

Adequately describing and handling the sources of variability is a challenge for performing robust ecotoxicology tests. Despite extensive validation efforts and improved statistical techniques, we must be pragmatic that some test systems and selected parameters will be highly variable, even under controlled conditions. In some cases, for example where vertebrates are concerned, it may not be possible to fully optimise designs for ethical reasons. Therefore, we need better evaluation tools to make the most of the data that we can collect.

### What are historical control data (HCD) and how could they help?

One route to contextualise the results obtained from a single study is to compare responses to previous studies performed under similar conditions (same methods and species), the so-called historical control data (HCD; Box 2). This is one of the benefits of regulatory test guideline studies being performed to standardised methods. Using established data sets of control group outcomes from historical studies can provide a greater understanding of the variability in the test system and therefore what is within the range of expected responses. It is therefore possible to improve the interpretation of a single study by adding context with no additional testing, as these data already exist.

#### Box 2 Historical control data

Historical control data are control data compiled from similar studies, performed either before or after the concurrent study. The basic assumption for using historical control data is that the past performance of test subjects under a particular set of conditions is a good predictor of future or previous performance. They can therefore be used, together with the concurrent control, to understand what a ‘normal’ response is for a particular type of test subject under a particular set of conditions, and therefore to help determine when a treatment response maybe outside the norm.

Historical control data are used in a variety of contexts. In human clinical trials, historical control data can mean more trial resources being devoted to the novel treatment and less need for the use of placebos/ineffective alternative treatments within a concurrent control (e.g., Viele et al. 2014). Historical control data are commonly used in mammalian toxicology studies, such as contextualising the occurrence and frequency of tumours in carcinogenicity studies (e.g., Deschl et al. 2002). Historical control data are also useful in engineering when determining normal tolerance intervals, for example when building very sensitive medical equipment (e.g., Young et al. 2016).

HCD are routinely used to aid the interpretation of mammalian toxicology studies for human safety assessment. In fact, in the EU the HCD are a legal requirement for active substance and metabolite toxicology studies for plant protection products (Section 5 of Regulation (EC) 283/2013): ‘*where available, historical control data shall be provided routinely. The data submitted shall be for endpoints that could represent critical adverse effects, and shall be strain-specific and from the laboratory which carried out the index study. They shall cover a five-year period, centred as closely as possible on the date of the index study*’. Within the same regulation, it goes on to further detail the types of information needed from the HCD set, e.g., type/source of test animals used, where/when the study was done, how the animals were maintained, age/size of animals at start/end of study, mortality observed (see Section 5.6 of Regulation (EC) 283/2013). Data are normally presented in a study by study basis; summary statistics (mean, median, standard deviation) are also usually provided. Interestingly, although there appears to be broad agreement in terms of what types of information and data should be included within a historical control database (e.g., Regulation (EC) 283/2013; Keenan et al. 2009), there is little guidance on how the data should subsequently be used when interpreting the results from a single study, though recommendations are available for particular types of studies, e.g., genetic toxicology tests (OECD 2017). For example, HCD is useful for setting a benchmark dose as a point of departure where responses are observed i.e., mammary tumours consistently observed in the controls (see Crump 1995).

In some disciplines, for example clinical trials, HCD is also incorporated into the statistical analysis of data (Pocock 1976). At the simplest level this can be to replace or supplement controls by historical controls. However, this can require rather precise assumptions about the similarity of conditions amongst datasets. Complex techniques can also be that down weight the historical information using different Bayesian priors methodologies (Neuenschwander et al. 2010). Such approaches are out of the scope of this manuscript.

Although the use of HCD is common place in toxicology, such an approach is not currently routinely used or even requested in ecotoxicology. Some contract research laboratories will report and use historical control data to aid the interpretation of ecotoxicity studies (personal observations of the authors), but typically only when an issue has arisen that needs resolving. One example of where historical control data are specifically mentioned for ecotoxicology is in the current guidance document for the assessment of risks to birds and mammals in the EU (Section 2.3.1(d) of EFSA 2009), where they can be used in the interpretation of avian reproductive studies. However, even though the use of HCD is written in the guidance, it is not routinely used to interpret findings in ecotoxicology.

The aim of this paper is to discuss the case for better use of HCD in ecotoxicology assessments, using case studies to show the use of HCD in the interpretation of a range of different studies. We hope that this will raise the issue in ecotoxicology and encourage organisations such as OECD and EFSA to develop guidance on how to perform such assessments on historical data, leading to greater use and regulatory acceptance. We believe this is not only a scientifically valid approach but also an ethical issue (Burden et al. 2015) that is in line with societally driven legal mandates to minimise the use of vertebrate testing in chemical regulatory decision making (e.g., Article 62 of the Plant Protection Products Regulation (EC 2009), Article 25 of the Registration, Evaluation, Authorisation & Restriction of Chemicals (REACH) Regulation (EC 2006), Article 62 of the Biocidal Products Regulation (EC 2012)).

## Case studies illustrating the value of historical control data

In the following case studies we illustrate how historical control data can be used to improve the interpretation of standard laboratory and higher tier studies. However, the case studies also raise several issues that highlight the need for better standardisation of approaches to facilitate better scientific and regulatory acceptance. In each case, we provide a brief overview of the purpose of the study and how historical control data could aid the interpretation.

### Standard laboratory studies

#### Avian reproduction studies

Avian reproduction studies are required to assess the risks to wild birds from potential exposure to plant protection products in many regions (e.g., EU, USA). These studies evaluate the reproductive effects of dietary exposure of adult birds (usually bobwhite quail or mallard duck) to a test substance over a twenty week period, usually performed to OECD 206 (OECD 1984) and/or OCSPP 850.2300 (U.S. EPA 2012a). The main focus is effects on reproductive parameters: number of eggs laid, fertility of the eggs, development of eggs including viability and survival of the embryos, hatchability, offspring survival, and egg shell thickness. Studies are performed throughout the year using birds sourced from bird suppliers that are acclimated to laboratory conditions. Birds are typically paired and randomly assigned to treatment groups. Pairs are housed individually in pens and fed either control diet or diet incorporated with the test item. Adults are monitored daily and their body-weights regularly measured in pre-egg laying (~10 weeks) and laying (10 weeks) phases. Laid eggs are assessed for egg shell thickness, cracks or any other abnormalities, and embryo mortality or infertility. Hatching is recorded and chicks are weighed immediately and after 14 days. All data and calculated indices of the response variables are analysed to determine statistically significant differences amongst treatment groups. A large amount of data are collected, including responses that might be expected to be highly variable (reproductive parameters).

Experience has shown that the issues postulated above, such as statistical differences between the concurrent control and treated groups potentially by chance alone and sometimes appearing to be treatment-related, are not uncommon.

The potential value of historical control data for interpreting avian reproduction studies was discussed in Valverde-Garcia et al. (2018). Historical control data were collated for avian reproductive toxicity studies undertaken over a 32-year period at a single contract research organisation (CRO), comprising 301 bobwhite quail and 292 mallard duck studies. Valverde-Garcia et al. (2018) proposed that, once collated, such an extensive historical control dataset could be used to contextualise the results from individual avian reproduction studies performed at the same CRO. For each reproductive endpoint, they calculated confidence and prediction limits of the means. The latter provides information about the expected value of the mean of a future study, and can be used to evaluate the reproductive performance of the birds used in a particular study relative to expectations under the same test conditions of that experiment in the context of the known HCD (see Valverde-Garcia et al. (2018) for further detail).

Two case studies were presented in Valverde-Garcia et al. (2018) that illustrated the interpretive value of HCD. The first provided an example of where exposure to the maximum tested dose appeared to result in a reduction in reproductive performance when compared to the concurrent control, though the difference was not statistically significant. However, when compared to the historical control data, the treatment mean was clearly within the realms of a ‘normal’ response, being within the prediction limits for the HCD. The second case study provided an example of where exposure to all tested doses appeared to result in a reduction in reproductive performance when compared to the concurrent control. However, when compared to the historical control data, the concurrent control mean was unusually high and all of the treatment means were within the realms of a ‘normal’ response, being within the prediction limits for the historical control dataset. Therefore, in both case studies, it is unlikely that the apparent reduced performance was treatment related, instead being due to natural variability.

The approach taken in Valverde-Garcia et al. (2018) can equally be applied to data sets from other studies types. The methods and time windows may need adjustment but such decisions if presented transparently can be justified for smaller datasets.

#### Fish full life cycle (FFLC) studies

Fish full life cycle (FFLC) studies are used to investigate the effects of exposure to a chemical on development, growth and reproduction of fish over a complete life cycle (Crane et al. 2010). Exposure usually begins with fertilised eggs (F_0_ generation), which are continually exposed to the chemical until the eggs have developed into adult fish (F_0_), a proportion of which are allowed to breed and produce eggs (F_1_), which are sometimes observed until the swim-up (early fry stage) or early life stage (juvenile stage). The most commonly used method for this type of study is described by U.S. EPA (1996), though other methods have been developed primarily to characterise potential endocrine disruption, e.g., the Medaka Extended One Generation (OECD 2015) and Zebrafish Extended One Generation (under OECD development) studies. A large number of parameters are typically measured (e.g., hatching success, fecundity, survival, weight, length, etc.), with some measured at multiple time points in one or multiple generations. Due to the long duration of the study, and the large number of parameters measured, a large dataset is obtained. Some of the parameters measured (e.g., fecundity), while frequently sensitive, are inherently variable, producing noisy data with relatively low statistical power but that is still considered fundamentally important (Crane et al. 2010). Interpretation of the data can therefore be challenging and the use of HCD may be a powerful tool to assist contextualising the results observed.

**Fig. 2:**
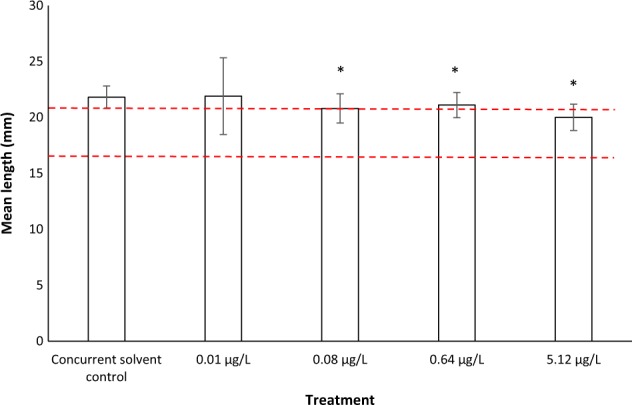
Effect of tamoxifen citrate (µg/L) on mean (±1 SE) length (mm) of F_1_ fathead minnow larvae. Asterisk denotes where mean is statistically significantly different to the concurrent solvent control (*p<* 0.05). Red dashed lines () indicate minimum and maximum mean length observed in historical solvent control data (based on 12 studies). [Figure created using data extracted from Williams et al. (2007)]

**Fig. 3:**
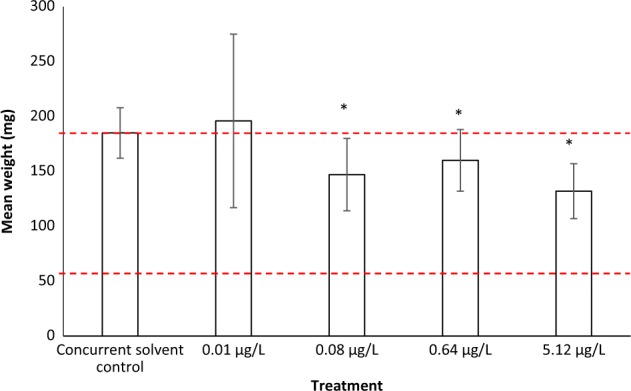
Effect of tamoxifen citrate (µg/L) on mean (±1 SE) weight (mg) of F_1_ fathead minnow larvae. Asterisk denotes where mean is statistically significantly different to the concurrent solvent control (*p<* 0.05). Red dashed lines () indicate minimum and maximum mean weight observed in historical solvent control data (based on 12 studies). [Figure created using data extracted from Williams et al. (2007)]

An example of such a case is described in Williams et al. (2007), in which the reproductive effects of tamoxifen citrate, a human pharmaceutical used for breast cancer treatment, to fathead minnow (*Pimephales promelas*) was investigated in a 284-day FFLC study, conducted to Good Laboratory Practice (GLP). Following exposure to tamoxifen citrate, statistically significant reductions in mean length and weight of F1 larvae (28-days post hatch (dph)) were observed in the 0.08, 0.64 and 5.12 µg/L treatments in comparison to the concurrent solvent control. As the study had been performed at a facility where many other similar studies were previously conducted, historical solvent control data were available. Using data extracted from Williams et al. (2007), the results obtained for F1 larvae length and weight are presented in Figs 2 and 3, respectively, together with the range of historical solvent control from 12 studies. Statistical significance as reported by Williams et al. (2007) is presented.

The mean lengths of F_1_ fathead minnow larvae in the 0.08, 0.64 and 5.12 µg/L treatments are slightly below (3.2–8%), and statistically significantly different, to that of the concurrent solvent control. However, in all three tamoxifen treatments, the mean lengths are within or slightly above the historical control range. The mean weights of F_1_ fathead minnow larvae in the 0.08, 0.64 and 5.12 µg/L treatments are also below (14–28%), and statistically significantly different, to that of the concurrent solvent control. However, in all three tamoxifen treatments, the mean weights are within the historical control range. The authors used the HCD, together with a lack of adverse effects on growth (F_0_ and F_1_ generation) and reproduction (F0) in adult fish, to conclude that the statistically significant reductions in growth of larval F_1_ 28-dph fish were not adverse effects, being within the realms of a ‘normal’ response. An overall NOEC of 5.12 µg/L was therefore concluded for the study using the HCD contextualisation, rather than a NOEC of 0.01 µg/L on the basis of purely statistical differences to the concurrent control.

The example provided by Williams et al. (2007) shows the potential value in using historical control data when interpreting a single study. However, little information regarding the historical control data set was provided in Williams et al. (2007). The authors refer the reader to a report (Williams et al. 2004) for further detail of the 12 studies that comprised the historical control dataset. However, Williams et al. (2004) is not publicly accessible, and therefore the details of these historical control studies (e.g. study design, test vessel volume, flow rates density of organisms, duration, solvents used, etc.) cannot be ascertained. It is therefore not possible for the reader to make a judgement on how comparable the historical studies are to the concurrent study, and therefore the relevance of the historical control data in terms of contextualising the concurrent study. This illustrates the need for HCD to be made available when being relied on for the interpretation of a particular study. In this context, clear guidance by appropriate authorities such as the OECD relating to the guidelines themselves or as a part of assessment procedures such as EFSA Guidance Documents would be useful.

#### Non-target terrestrial plant (NTTP) studies

Historical control data are not only useful in interpreting where an effect is outside the realms of ‘normal’, but also in determining what level of effect is likely to be detectable given the extent of natural variation in the test organism response. A good example of this was recently presented by Stavely et al. (2018) for non-target terrestrial plants (NTTP). NTTP studies are used to assess the potential effects of chemicals, such as plant protection products, on vegetative vigour and seedling emergence. Vegetative vigour studies assess the effects of chemical exposure on post-emergent plants, and are usually performed to OECD 227 (OECD 2006a) or US EPA 850.4150 (U.S. EPA 2012b) guidelines; seedling emergence studies assess the effects on emergence and growth, and are usually performed to OECD 208 (OECD 2006b) or U.S. EPA 850.4100 (U.S. EPA 2012c) guidelines.

Guideline methods were originally designed to estimate the application rate causing a 25% effect (ER_25_, used in the USA) or 50% effect (ER_50_, used in the EU) on vegetative vigour or seedling emergence. However, there is now a move towards requiring no observed effect rates (NOER) or, in their absence, lower ER_x_ values e.g. ER_5_ (proposed for the U.S. (U.S. EPA 2013)), ER_10_ (proposed for the EU (EFSA 2014)). Although values of ER_5_ and ER_10_ can be estimated where a regression model has been fitted to the dose-response data (by extrapolation rather than interpolation), these estimates may not be statistically reliable and therefore not useful. Stavely et al. (2018) investigated whether it was possible to reliably detect a 5 or 10% difference compared to controls given the natural variability in responses, and if not, what level of difference to controls could be reliably detected.

HCD from approximately 100 studies on vegetative vigour and seedling emergence undertaken during 2004–2015 were collated, provided by 9 different chemical companies, undertaken in 13 different laboratories encompassing 53 pesticide test substances. All studies were conducted according to GLP and the relevant OECD or USEPA guidelines. For each study, various factors were recorded within the database that might influence the results obtained e.g. test facility, year study was initiated, duration, species, etc (see Stavely et al. (2018) for further detail). For both types of study (vegetative vigour and seedling emergence), the main growth parameters of shoot height and dry weight were collated.

To assess the variability within the historical control dataset, coefficients of variation (CVs) and minimum detectable differences (MDD%) were determined for each species, for each growth parameter (shoot height and dry weight) for both seedling emergence and vegetative vigour. The MDD% is analogous to a power calculation; for example, if MDD% = 20, then it is unlikely that an effect of <20% can be detected/estimated, and it is likely that an effect of >20% can be detected/estimated. For each species, test type and growth response combination, the distribution of CVs and MDD%s was determined, including the mean, median, 25th and 75th quantiles, together with minimum and maximum values. In terms of deciding whether an effect level (i.e., ER_x_) was reliable, it was considered that the 75th quantile of the MDD% (MDD%75) should be less than the stated effect level, e.g., if the MDD%75 was 7, then an ER_10_ would be reasonable to use in the risk assessment, but an ER_5_ would not.

The variability observed in the HCD for each species, study type and growth parameter was summarised. Data on dry weight in the control tended to be more variable than shoot height data in both study types, and variability was higher in seedling emergence studies than vegetative vigour studies. The MDD% was rarely below 5 for all growth parameters and study types, suggesting that an ER_5_ is not appropriate for NTTP studies. This was confirmed when the MDD%75 values were compared with the desired effect level (ER_x_). In all cases, a 5% effect (ER_5_) cannot be reliably detected, as all MDD%75 values were >5. The situation for estimating 10% effects (ER_10_) was only slightly improved, with reliable estimates being possible in 12% of cases. For 25% effects (ER_25_), the results were more encouraging, with reliable estimates being possible in 82% of cases.

The historical control data analysis indicates that if ER_5_ or ER_10_ values are to be used in NTTP risk assessments, then the variability within these studies would need to be dramatically reduced; however, the options for doing so are limited. Unlike for vertebrates, there is no legal obligation for minimising the number of replicates or test item rates tested for NTTPs; however, there is a practical limitation. The current study design of vegetative vigour and seedling emergence studies already requires a large amount of space due to the number of species tested, together with the numbers of rates used and replicates for each rate and species combination. Therefore, as concluded in Stavely et al. (2018), it is more practical to use the more reliable ER_25_ estimates in the risk assessment, and instead think about how these can be incorporated to achieve the desired level of protection. This example nicely demonstrates how HCD could be used to help translate protection goals into reliable and achievable regulatory targets, taking into account what is desired versus what is practically possible and statistically reliable.

### Higher tier studies

#### Mesocosms

When moving from single species laboratory studies to more realistic, semi-field community studies, such as aquatic mesocosm studies, the level of complexity of the data exponentially increases with the numbers of organisms involved and the variation in environmental parameters. Aquatic mesocosms aim to mimic edge-of-field waterbodies and are used to assess realistic risk of plant protection products (PPPs) to aquatic communities as part of the higher tier aquatic risk assessment for PPP authorisation (EFSA 2013). The increased realism of these studies, reduces uncertainty when extrapolating effects to the natural environment; however, the statistical power, and thus the reliability of an observed effect, are impaired by the high intrinsic variability in these highly dynamic and complex systems. To address this in the EU, the current aquatic guidance (EFSA 2013) has introduced the use of minimal detectable differences (MDDs) as a reverse power analysis to enhance certainty and interpretability of these studies that often have low replication (due to costs and practicalities) and high control and within treatment variability.

HCD can be useful in characterising mesocosm study results. In mesocosm studies, whilst the controls are not treated, they are not controlled systems as in laboratory studies. By design, these controls are similar to natural systems and a high number of biotic and abiotic factors interact, with these interactions changing over time. HCD can provide invaluable information regarding the seasonal dynamics of the system and to highlight which abiotic and/or biotic variables affect the control populations the most. When statistically significant differences are determined between the control and treatment replicates, these can then be discussed on the basis of the knowledge from the HCD and support decision making. This can help to avoid false positives or negatives in terms of test item effects and add to a more comprehensive understanding of the test system.

To demonstrate how HCD can be used to help distinguish between treatment-related effects and normal seasonal dynamics, control group data were collated from twelve mesocosm studies performed between 2011 and 2017 at Cambridge Environmental Assessments’ mesocosm facility (Cambridge; UK), comprising 48 control replicates covering eighteen zooplankton taxa and five environmental variables. A generalised additive model (R 3.5.1 (2018-07-02)—“Feather Spray”: packages ggplot2 and mgcv; GAM) was used to investigate the influence of environmental parameters and seasonal dynamics on the zooplankton community.

**Fig. 4:**
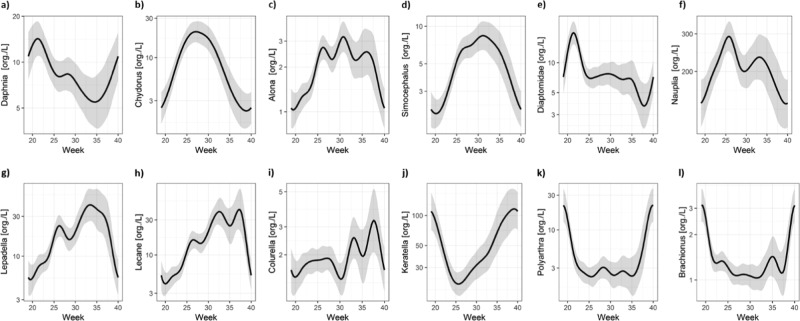
Zooplankton abundances during the course of a mesocosm season using data collated from control replicates in 12 mesocosm studies: **a**–**f** different Cladocerae; **g**–**l** different Rotiferae; species names given on y-axes; Mesocosm season starts in Week 19 = ~10th May; Mesocosm season ends in Week 40 = ~4th October. Solid black line = fit from generalised additive model (GAM); grey shading indicates 95% confidence interval

Abundances of individual zooplankton taxa are driven by abiotic environmental parameters (e.g. temperature, pH, dissolved oxygen and conductivity) as well as biotic factors through competition or other biological processes such as food availability (e.g., chlorophyll) and predation. This is reflected in the abundance patterns observed throughout the season, with Cladocera species (Fig. 4a–f) generally being more abundant during the spring and early summer whereas Rotifera (Fig. 4g–l) appear from mid-summer onwards. Larger zooplankton (such as Cladocera) have been reported to be more sensitive to insecticides compared to the smaller taxa (e.g., Hanazato 1998; Clements and Rohr 2009). As Cladocera are generally more abundant early in the season, exposure to insecticides applied in spring could therefore accelerate the natural seasonal succession from larger to small zooplankton, by reducing competition with larger zooplankton following PPP exposure. However, this also means that natural succession induced by a change in environmental parameters could be misinterpreted as a test item effect; for example, according to the HCD analysis, non-treatment related increases in pH or dissolved oxygen also result in reduced abundances of larger zooplankton and in turn, competitive release of smaller zooplankton. HCD are therefore invaluable in understanding the interaction between taxa abundance and environmental parameters, and therefore understanding the cause of the effect and avoid misinterpretation.

**Fig. 5:**
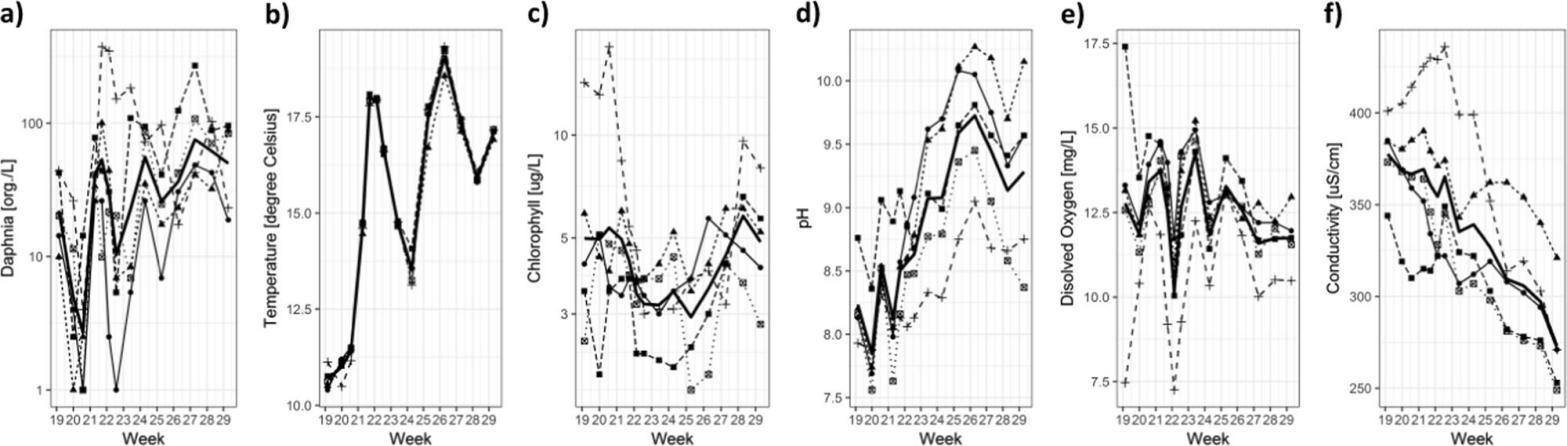
Control data from a single mesocosm study: **a***Daphnia* abundance, **b** temperature, **c** chlorophyll, **d** pH, **e** dissolved oxygen, **f** conductivity. Mean = bold black line, Individual replicates = lines with symbols

Historical control data can also be helpful in interpreting within treatment differences. The GAM showed that high abundance of *Daphnia* usually coincides with the start of a mesocosm study (Week 21; Fig. 2a), together with low Chlorophyll concentrations, low pH and high conductivity. However, sometimes replicates within mesocosm studies do not follow the ‘normal’ trends, which could be misinterpreted as test item effects. In the following example (Fig. 5), *Daphnia* mean abundance in the control replicates initially decreased at the start of the mesocosm study (Week 20), and then increased until Week 30 i.e. opposite to the HCD trend. On closer inspection, the replicate driving the high *Daphnia* abundance was related to abnormal levels in abiotic factors (high conductivity, low pH, low dissolved oxygen; Fig. 5). Such a relationship between abiotic factors and *Daphnia* abundance is in agreement with the findings of the HCD analysis. Outliers within a treatment group can therefore be explained using the knowledge obtained from the HCD regarding the impact of environmental parameters or competing species on specific taxa.

It is clear that a detailed knowledge of the underlying test system is essential to distinguish between a change in abundance being due to either changes in biotic/abiotic factors or to test item exposure. HCD are clearly a valuable tool for the interpretation of mesocosm studies, providing context for how environmental parameters influence individual populations, and also indicating which species are most likely to co-occur. Clearly a comprehensive database is essential, but to achieve this a number of studies need to be available and the database to be continually updated, which is consequently a long-term approach as mesocosms are commonly only conducted once a year.

## So why aren’t we using HCD in ecotoxicology?

It is clear from the case studies presented here that there is a valuable role for the use of HCD for the interpretation of ecotoxicity studies, so why aren’t they being regularly used at the moment? If they are routinely provided and considered in clinical trials and mammalian toxicology studies, and thus human health assessments, why not for ecotoxicology and wildlife assessments? Are there differences between ecotoxicology and toxicology studies, or other concerns, that prevent the use of HCD or limit their relevance? Often cited reasons are inherent differences in the parameters assessed and the potential for HCD to add noise into parameter interpretation that may mask real effects and a lack of guidance for the implementation of HCD approaches.

There are some types of data collected in mammalian toxicology studies that are inherently ‘different’ to ecotoxicology studies. As toxicology studies are done primarily to assess individual level effects, there are various endpoints that are not typically measured in ecotoxicology studies e.g. incidents of tumours (Deschl et al. 2002) and malformations (Mylchreest and Harris 2013). For these types of effects, where occurrence in control organisms maybe rare, the value of a larger data set to determine whether the frequency of occurrence in the concurrent study is normal or not is a very powerful tool. However, these are not the only data types for which HCD is collected; indeed, Section 5 of Regulation (EU) 283/2013 states ‘…*where available, historical control data shall be provided routinely. The data submitted shall be for endpoints that could represent critical adverse effects…’*. Therefore, HCD are collated for whichever parameters are critical in driving the NOEL, including endpoints that would also be relevant for the protection of wild mammal populations (see Crane et al. 2018).

When citing HCD to contextualise differences observed in the concurrent study, there can be concerns from regulatory authorities that using HCD could mask treatment-related effects. However, the counter argument to this is that these ‘effects’ aren’t real, and are just a reflection of the natural variability within a test system and their ecological relevance is questionable. If there is a desire from regulatory authorities to be able to reliably detect smaller effects, then there would need to be a review of the test methods currently used in regulatory studies. To achieve this aim, efforts would be needed to artificially reduce the uncontrollable variability in response parameters (Fig. 1). For instance, by using organisms of reduced genetic diversity (such as inbred strains used in mammalian toxicology or further use of clonal organisms, e.g., *Daphnia*). However, such choices may not be desirable in terms of the representativeness of test organisms to those exhibiting greater diversity in the environment requiring protection. Perhaps more tractable would be attempts to address animal husbandry and identify design elements important to the ‘controllable’ variability (Fig. 1). Undoubtedly, systemic improvements could be made that would lead to reduced variability. However, it is likely that the most improvement could be achieved by increasing replication or the number of treatment levels (depending on statistical design). As discussed in the examples above, the ethical and practical limitations of performing studies to optimised statistical precision will often be the limiting factor. We would argue that an understanding of historical control data performance of intra- and inter-laboratory variability would inform the best development of new and continuous improvement to existing study guidelines. To this end, we need to better understand the factors contributing to the variability in response for each study type and species, e.g., source of organisms, environmental factors, etc, to identify those factors which have the biggest influence on variability and therefore which should be considered when assessing the relevance of historical control data.

Perhaps the most limiting factor for the use of HCD in ecotoxicology assessments is the lack of guidance in terms of how to use it, what data are relevant to include, when they are relevant, how to compare the HCD to the concurrent study, etc. Such clear guidance of how to use HCD also does not exist for toxicology assessments, but this has not prevented the data being used. Proposals for how to use HCD in an ecotoxicology perspective have been made (e.g., Valverde-Garcia et al. 2018), but these need to be applied to other ecotoxicological study types and species, and evaluated and formalised by organisations such as OECD and EFSA.

## Conclusions and recommendations

The case studies presented here illustrate how HCD can be useful in contextualising the results from a single study. It can also be useful when determining what level of effect (e.g. ECx) may be most meaningful for a given parameter in a test system. As such an understanding of the HCD can lead to improvement of test designs, better linkage of laboratory results/interpretation to the protection goals, make the best use of all available data and avoid unnecessary additional data generation (particularly for vertebrates) or the need for extensive higher tier risk assessments. We believe this is one of the strongest motivators for the routine collection and use of HCD. We hope these benefits challenge the perception that laboratories and sponsors are only interested to use HCD to raise no effect levels in individual studies.

HCD can be particularly useful for study designs with few treatment levels and low numbers of replicates, typical of sublethal effect vertebrate studies, endocrine screening studies and higher-tier studies such as mesocosms. To increase the number of treatment levels and/or replicates for such studies would increase vertebrate testing and/or be prohibitively expensive. It also makes best use of data that has already involved vertebrate animal use so that the maximum information is extracted considering ethical implications of generating the data. HCD can also be useful in situations where a study has been undertaken and a decision to repeat the study or undertake further studies needs to be made. Therefore, the approach can be useful for any standardised ecotoxicology study design.

We can make more use of the wealth of data that are available by looking at the bigger picture. One of the advantages of using standard study designs and species in regulatory toxicity tests is that the results from control groups amongst studies are comparable. This can also be the case for non-standard higher tier studies if there is a sufficient body of data available from historical studies performed with comparable methods and environmental conditions.

We recommend that historical control data should be collated, so that the results from single studies can be contextualised. To allow for confident comparisons, information on the conditions under which the historical studies were performed should be provided, so that environmental conditions and pertinent test system design elements can be considered. The requirements of historical control data from toxicology studies could be used as a guide for which information are important to include. To this end, we hope organisations such as OECD, EFSA and USEPA develop guidance on the principles of historical data collection, reporting, statistical analysis and interpretation to aid and encourage the use of these valuable data in ecotoxicity study design and interpretation.

Development of robust and reliable historical control databases should also be used to support the regular update of study guidelines that would support improved outcomes based on an understanding of acceptance response ranges. Levels of variation can change over time as study methods change/improve, genetic drift in laboratory animals occurs, etc. We would encourage the organisational bodies that are responsible for the development and revision of study guidelines (e.g., OECD) to regularly revisit their guidelines to evaluate whether performance criteria or other elements need adjusting. Such an activity was recently undertaken as part of the Fish Toxicity Testing Framework (OECD 2014), with revised technical guidelines being developed as a result of publications analysing HCD of regulatory studies (e.g., Oris et al. 2012).

We hope this article will encourage the debate on the appropriate use of HCD in the interpretation of ecotoxicology studies such that we can use all the tools at our disposal to improve environmental decision making in a scientifically robust but resource responsible manner.
